# The Olfactory Tract: Basis for Future Evolution in Response to Rapidly Changing Ecological Niches

**DOI:** 10.3389/fnana.2022.831602

**Published:** 2022-03-03

**Authors:** Kathleen E. Whitlock, M. Fernanda Palominos

**Affiliations:** ^1^Centro Interdisciplinario de Neurociencia de Valparaíso (CINV), Universidad de Valparaíso Valparaíso, Chile; ^2^Instituto de Neurociencia, Universidad de Valparaíso Valparaíso, Chile

**Keywords:** gonadotropin releasing hormone (GnRH), immune system, neutrophils, climate change, limbic system, teleost fishes

## Abstract

Within the forebrain the olfactory sensory system is unique from other sensory systems both in the projections of the olfactory tract and the ongoing neurogenic potential, characteristics conserved across vertebrates. Olfaction plays a crucial role in behaviors such as mate choice, food selection, homing, escape from predators, among others. The olfactory forebrain is intimately associated with the limbic system, the region of the brain involved in learning, memory, and emotions through interactions with the endocrine system and the autonomic nervous system. Previously thought to lack a limbic system, we now know that teleost fishes process emotions, have exceptional memories, and readily learn, behaviors that are often associated with olfactory cues. The association of neuromodulatory hormones, and more recently, the immune system, with odor cues underlies behaviors essential for maintenance and adaptation within natural ecological niches. Increasingly anthropogenic perturbations affecting ecosystems are impacting teleost fishes worldwide. Here we examine the role of the olfactory tract as the neural basis for the integration of environmental cues and resulting behaviors necessary for the regulation of biotic interactions that allow for future adaptation as the climate spins out of control.

*“I should think we might fairly gauge the future of biological science, centuries ahead, by estimating the time it will take to reach a complete, comprehensive understanding of odor. It may not seem a profound enough problem to dominate all the life sciences, but it contains, piece by piece all the mysteries.”*

*—Lewis Thomas (1985)*.

## Introduction

As highlighted by Thomas (1985) the understanding of odor is a puzzle that encompasses, “piece by piece all the mysteries of life”. In comparison to other sensory systems, odor cues are processed differently in the brain, bypassing the thalamus and projecting directly to cortical regions of the brain involved in learning, memory motivation, and emotion (Carpenter, 1985). Olfaction, unlike vision or hearing, is a sensory system that relies on signals that persist in the environment and are transmitted by complex plumes in both air and water (Pannunzi and Nowotny, 2019). Odor signals provide essential information for biotic interactions within an ecological niche (Draper and Weissburg, 2019). In the last decade, it has become irrefutable that human-induced climate change is wreaking havoc on ecosystems around the world (Masson-Delmotte et al., 2021), including aquatic ecosystems. In considering the impacts of climate change there are two philosophies: the “egocentric” view where we prioritize the devastating effects of climate change on humanity (for example food production) and the more “ecocentric” view where we prioritize how human activities are destroying the web that connects all life. Here we will introduce the olfactory system of teleost fishes, emphasizing that they have homologous circuitry that corresponds to the limbic system in mammals. Next, we will highlight two aspects of the olfactory system: the neuroendocrine cells of the terminal nerve that contain a peptide unique to non-mammalian animals including teleost fishes, and the immune system as a basis of olfactory recognition, highlighting their roles in mediating biotic interactions within an ecological niche. Finally, we explore data suggesting that the olfactory circuitry essential for the interactions of fishes with their environments can adapt to the increasing climate-based interference of odor cues with hopes that we will not lose these fascinating animals with whom we share the web of life.

## The Forebrain

The forebrain of vertebrate animals is composed of the cerebrum, thalamus, hypothalamus, pituitary gland, limbic system, and importantly for this review, olfactory bulbs. In humans the forebrain, also referred to as the prosencephalon (Greek for the forward brain), is the largest region of the brain and can be subdivided into the telencephalon (olfactory bulbs, cerebral cortex, hippocampus, basal ganglia, and some portions of the limbic system) and the diencephalon (thalamus, pituitary gland, optic chiasm, mammillary bodies, and hypothalamus). One of the dominant functions of the forebrain is to process olfactory information (Mori and Sakano, 2021). Important when considering the processing of olfactory information in the forebrain is the limbic system, which in mammals includes primarily the amygdala and hypothalamus (Sokolowski and Corbin, 2012) although the limbic system is more generally described as where subcortical structures meet the cerebral cortex (Morgane et al., 2005). Originally called the rhinencephalon (meaning “smell brain”) because it receives considerable input from olfactory sources and was thought to be primarily involved with the sense of smell, we now know that the forebrain limbic system integrates sensory information to generate behavioral responses to stimuli within social, emotional, or motivational contexts (Morgane et al., 2005; Mori and Sakano, 2021). These include innate behavioral and emotional responses needed for survival: examples being mating, aggression, and defense (Gerlach and Wullimann, 2021; Mucignat-Caretta, 2021). Thus the forebrain is an essential link between odor cues in the environment and the olfactory circuits that generate behaviors necessary to adapt to a given ecological niche over time.

### Teleost Brains

Approximately 50 percent of vertebrates species on the planet are fishes and of this group almost 95% are teleost fish (Pough et al., 2005), a group of animals that have adapted to extreme ecological niches, ranging from the deep oceans in the absence of light and under great pressures to “walking on land” as in the case of mudskippers who live both in and out of water. In general, teleosts have small brains relative to body size although once again they show great diversity across species; whereas most vertebrate species have similar brain-to-body mass ratios, fishes are unusual in the extreme variation of the brain to body mass ratios; the deep-sea bony eared assfish (*Acanthonus armatus*) has the smallest ratio known in vertebrates (Fine et al., 1987) and the elephantnose fish (*Gnathonemus petersii*), a freshwater species found in Africa, has one of the largest brain-to-body weight ratios (Nilsson, 1996), even slightly higher than that of humans. Thus this great diversity in brain size and ecological niches suggests that structural (neural) and concomitant behavioral plasticity in teleost fishes have allowed this group of animals to rapidly adapt to past environmental pressures influencing biotic interactions within an ecosystem.

Like all sensory systems, the size of neural tissue within the central nervous system (CNS) dedicated to a given sensory modality reflects the importance of that modality to the behavior of the animal. Thus animals that rely heavily on olfactory cues, for example, have a distinctly different proportion of “brain space” dedicated to that modality: humans with approximately 50% of the genes coding for olfactory receptors existing as pseudogenes have much smaller olfactory bulbs relative to a mouse with approximately 1,000 expressed olfactory receptors (Niimura and Nei, 2005). In fishes this is evident in the size of the peripherally located olfactory epithelia where in general having more lamellae is correlated with an increased number of genes coding for olfactory receptors (Policarpo et al., 2021). Furthermore, the olfactory organs of fishes are structurally diverse with differences in the distribution of sensory neuronal subtypes within the epithelia (see for review Kasumyan, 2004). In general, the dedication of “neural space” to the detection and processing of odorants reflects the great importance this sensory system has for fishes in general.

**Figure 1:**
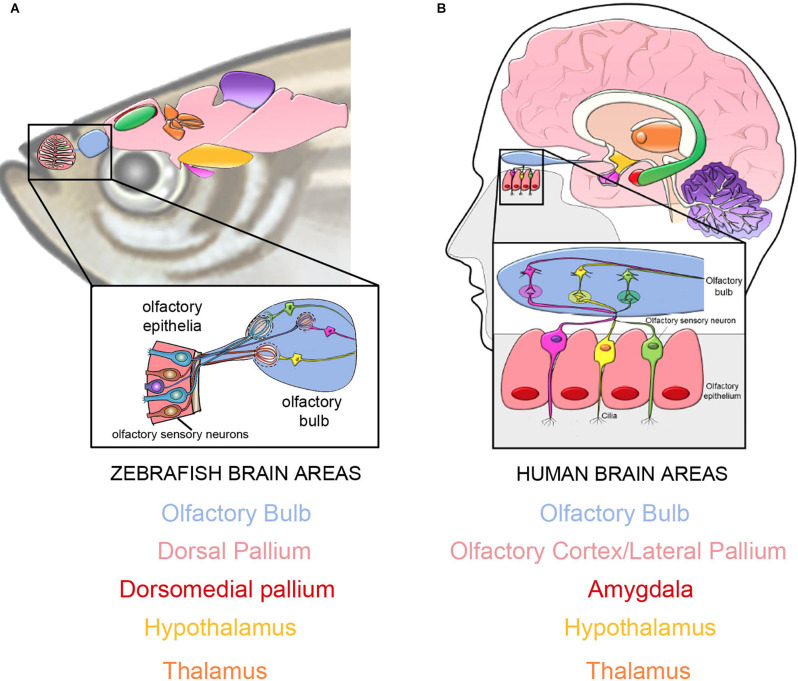
The connections from the peripheral olfactory epithelia to the olfactory bulbs are highly conserved in vertebrates. In both in teleost fish (**A**, zebrafish: modified from–Calvo-Ochoa and Byrd-Jacobs, 2019) and humans **(B)** the OSNs relay information to the olfactory bulbs (blue) continuing to the dorsal pallium in fishes **(A)**, and the olfactory cortex/lateral pallium **(B)** in mammals, thus bypassing the thalamus (orange). Both species have projections from the olfactory bulbs (blue) to the amygdala (red, **B**) and its proposed equivalent in teleosts, the dorsomedial pallium (red, **A**).

Originating from cells organized along the anterior posterior axis of the developing embryo, the telencephalon of jawed vertebrates have similar identifiable regions (Figure 1) where differences among vertebrate classes can be attributed to morphogenic movements as well as altered rates of proliferation. In teleosts, the brain arises through morphogenetic movements, distinct from tetrapods, where the telencephalon arises through eversion of the dorsal aspect of the neural tube (Nieuwenhuys, 2009). This results in a single ventricle flanked by neural tissue where the main pallial target of the olfactory bulb in teleosts, the dorsal pallium (Dp: homologous to lateral pallium in tetrapods) lies in a ventrolateral position (Puelles López et al., 2008; Bruce and Braford, 2009).

In contrast to the original proposal that (teleost) fish lack the prerequisite neural architecture for phenomenal consciousness including pain, and thus cannot feel pain (Key, 2015), studies have refuted this statement at the molecular (Sneddon, 2019), behavioral (Sneddon et al., 2003; Braithwaite and Boulcott, 2007) and circuital/anatomical level (Maximino et al., 2013). Furthermore, recent results showed that the cleaner wrasse (*Labroides dimidiatus*) can pass a mirror-mark test, where the animal is able to use a mirror to recognize a mark on its own body (Kohda et al., 2019). Although controversial (de Waal, 2019), these results suggest that the conscious awareness of oneself as distinct from the world outside may be a characteristic of many different animals, including fish. This is an important issue when considering the olfactory tracts in the forebrain because in terrestrial vertebrates the limbic system is responsible for the emotional behaviors triggered by olfactory cues. Thus the potential “limbic equivalent”, the dorsomedial pallium and hypothalamus as well as other brain nuclei, in teleosts receives input from olfactory sources in addition to input derived from many other areas. Accepting that fishes have neural pathways similar to the limbic system in mammals, and perhaps some grade of “consciousness” (Kohda et al., 2019; Birch et al., 2020), allows for the contemplation of a more complex level of neural processing in response to odor cues.

## Olfactory System

### Structures

The basic organization of the olfactory sensory system is highly conserved across vertebrates (Ache and Young, 2005), where odors are detected by the primary olfactory sensory neurons (OSNs) located in the neural epithelia of the olfactory organ. The axons of the OSNs form the olfactory nerve that will cross the cribriform plate and make their first synapses in the highly organized olfactory bulb, the first site of information processing. The olfactory tract, extending centrally from the OBs is unique among sensory systems because the sensory information does not pass through the thalamus en route to the piriform cortex where the olfactory sensory information is further processed. Additionally, the olfactory tract also projects to the amygdala and hypothalamus, part of the limbic system, as well as a number of other targets in the forebrain. Thus the first “relay” within the olfactory tract is composed of the peripheral olfactory epithelium (OE), the olfactory nerve (ON) that conducts the information, and a central target, the olfactory bulb (OB; Whitlock, 2006, 2015; Friedrich et al., 2010; Kermen et al., 2013).

### Development

Like other sensory systems the olfactory placodes that give rise to the olfactory epithelia, are proposed to be induced from the ectoderm placodes (Schlosser, 2010; Aguillon et al., 2018). This model does not work as well for the olfactory sensory system. Unlike taste or hearing where the sensory receptors (taste buds, ear hair cells) have their first synapse in the periphery, the primary OSNs that detect odors are also the same neurons that extend axons into the central OBs, where the initial synapse is made. Thus the placode model separates the development of the olfactory sensory neurons from that of the adjacent neural tube from which their central targets arise. Previously we have shown in zebrafish that olfactory placodes arise from a large field of neurectodermal cells continuous with the telencephalic precursors in the neural plate and that cell movements, not cell division, underlie olfactory placode morphogenesis (Whitlock and Westerfield, 2000; Whitlock, 2008). These studies lead to a model whereby a continuous neurectoderm generates both the OBs and olfactory placodes, thus coordinating peripheral OSNs and their central targets during developmental and evolutionary time (Torres-Paz and Whitlock, 2014; Torres-Paz et al., 2021). This suggests that when faced with external selective pressures, adaptive responses would act on the OSNs and their central synaptic targets as a single developmental and functional unit.

## GnRH Cells: Neuromodulatory Network Originating in The Olfactory Forebrain

The terminal nerve (TN), or nervus terminalis is one of the most enigmatic neural pathways in the vertebrate forebrain. It was the last discovered of the cranial nerves and is referred to as Cranial Nerve 0 or XIII. This vertebrate-conserved multifaceted nerve, in some species, appears to influence sensory processing, sexual behavior, and autonomic and vasomotor control (Wirsig-Wiechmann et al., 2002). The TN has fibers that originate from cell bodies associated with the anterior olfactory tract but has a highly species-specific projection pattern within the forebrain. There is ample discussion as to the defining characteristics of the TN, and the presence of gonadotropin-releasing hormone (GnRH)-immunoreactive neurons appears to be one defining feature. Teleost fishes have a population of neurons expressing the fish-specific GnRH3 isoform in the TN, and this population is thought to play a neuromodulatory role in multiple physiological systems, including olfaction, vision, and reproduction.

### Origin of TN

The TN has diverse embryonic origins that may shed light on its function(s) in the adult animal. Analysis of the development of the GnRH cells associated with the olfactory sensory system supports a neural crest origin based on lineage tracing in zebrafish (Whitlock et al., 2003) and mice (Forni and Wray, 2012) yet a subsequent study in zebrafish (Aguillon et al., 2018) concluded a homogenous origin from progenitors based on Islet-1/2 expression in all developing GnRH neuroendocrine cells. Subsequent studies showed that there are in fact two populations of GnRH cells: Islet-1/2+ and Islet-1/2−, thus consistent with two separate origins of GnRH neuroendocrine cells (Shan et al., 2020). The TN-GnRH3 neurons are the first populations of GnRH neurons to develop in the early embryo (Gopinath et al., 2004) where studies using fluorescent reporter lines and electrophysiology recording, have shown that these early differentiating TN-GnRH3 neurons acquire an adult-pattern of spontaneous action potential firing as early as three days post-fertilization (Ramakrishnan et al., 2010). Thus the TN-GnRH3 cells are early differentiating neuromodulatory cells associated with the developing olfactory tract.

In the adult animal, in contrast to the hypophysiotropic GnRH1 neurons in the preoptic area that show episodic spontaneous electrical activities, the TN-GnRH3 neurons show regular intrinsic pacemaker activities (Oka and Matsushima, 1993; Oka, 2010; Zhao et al., 2013). In adult fish (dwarf gourami) GnRH fibers originating from the TN cells are distributed widely throughout the brain, and to date, there is little evidence to support an olfactory-pituitary connection, thus suggesting that the TN-GnRH system most likely acts as a neuromodulator, capable of affecting widespread regions of the brain (Oka and Matsushima, 1993). The extensive projections of the TN-GnRH neurons in the forebrain, coupled with their endogenous rhythmic activities, suggest they may act in the global modulation of circuits to accommodate changes in the animal’s hormonal or environmental conditions (Umatani and Oka, 2019).

### TN-GnRH3 Neurons and Olfactory-Driven Behaviors

Pacific salmon show an amazing ability to migrate long distances returning from the ocean to their natal rivers to spawn. This behavior is based in part on the formation of an olfactory memory (olfactory imprinting) during early development (Scholz et al., 1976). At specific stages of salmon migration, GnRH peptides show dynamic patterns of expression in the brain (Ueda, 2012) where GnRH3 (originally called salmon GnRH) has been reported in the olfactory nerve of masu salmon (*Oncorhynchus masou*, Kudo et al., 1994) as well as the chum salmon (*Oncorhynchus keta*) when the animals were in the coastal waters, the region where olfactory decisions become important, but not in fish on the spawning ground (Kudo et al., 1996). Subsequent studies using fluoroimmunoassays (Yamada et al., 2002), have shown increased levels of GnRH3 correlating with the migration behavior; OBs in the salt to freshwater transition areas and the telencephalon within the river system, both regions where olfactory discrimination of natal river odors is essential (Ueda, 2011). These studies suggest that the TN-GnRH3 network as well as other regions of the forebrain participate in neuromodulation in the olfactory system and thus play important roles in salmon homing migration (Ueda and Yamauchi, 1995; Ueda, 2011, 2012).

Interestingly, there is evidence that olfactory cues can directly modulate the activity of the retina *via* the olfacto-retinal-centrifugal (ORC) pathway where the TN in the OBs extends to and terminates in the neural retina, a pathway that contains GnRH3 and FMRFamide peptides (Münz et al., 1982; Stell et al., 1984; Behrens and Wagner, 2004). Using the white perch (*Roccus americana*), it has been shown that within the retina GnRH stimulates the release of dopamine from the interplexiform cells while FMRFamide suppresses some effects of GnRH (Umino and Dowling, 1991). The ORC pathway, when probed at the behavioral level, interacts with olfactory cues: if first exposed to a food or a conspecific alarm odor, adult zebrafish are able to respond to a moving visual stimulus at lower light levels (Stephenson et al., 2012). This behavioral response, in zebrafish, has been proposed to be regulated by TN-GnRH3 axon projections that target the retina of the eye (Maaswinkel and Li, 2003). Thus the current model suggests that stimulation of OSNs through the ORC pathway alters the cellular activity of the TN (potentially by GnRH3 neurons) thus modulating retinal neural function by increasing visual sensitivity.

### GnRH3 Neurons and the Olfactory Epithelia

**Figure 2:**
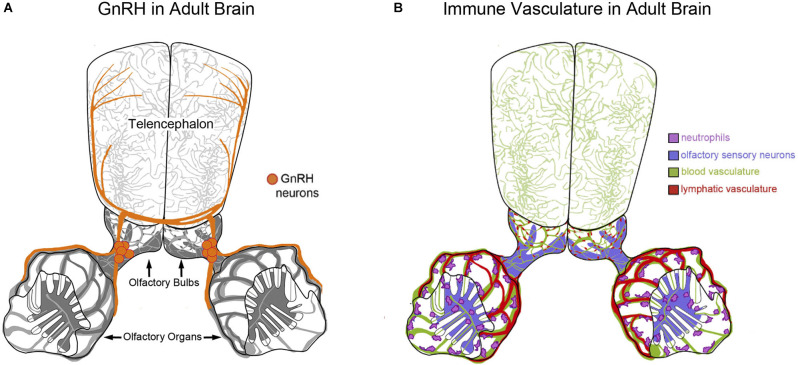
Neuroendocrine and immune factors that potentially modify olfactory function. **(A,B)** Diagram of the brain of adult zebrafish depicting olfactory organs and telencephalon. **(A)** Gonadotropin releasing hormone 3 (GnRH3) containing neurons (orange) of the terminal nerve are found intimately associated with the blood/lymphatic vasculature of the olfactory organ. **(B)** Neutrophils (pink) are associated with the extensive blood/lymphatic vasculature system (green/red) wrapping the olfactory organs as well as OSNs within the olfactory epithelia (purple) in zebrafish (Palominos et al., 2021).

Paradoxically, in spite of numerous labs searching for GnRH-positive nerve terminals in the olfactory epithelia there is to date no conclusive evidence for this type of direct neural connection. In teleosts fishes GnRH3-positive TN fibers have been localized to the lamina propria lining the olfactory epithelium in dwarf gourami (*Trichogaster lalius*, Wirsig-Wiechmann and Oka, 2002) and underneath the olfactory epithelium in goldfish (*Carassius auratus*, Kawai et al., 2009). Likewise in mammals, while no labeled GnRH-positive fibers have been observed within the olfactory and vomeronasal epithelia, conspicuous GnRH positive terminals were reported on blood vessels of the olfactory mucosa leading to the suggestion of a new neurohaemal area (Zheng et al., 1988). In spite of these intriguing observations, few studies have examined the potential for a neural-vascular or peripheral “olfactory neurohaemal organ” within the olfactory sensory system. In zebrafish we have recently described the blood/lymphatic vasculature in the developing olfactory organ (Palominos and Whitlock, 2021; Palominos et al., 2021) that runs adjacent to the region we previously described as containing cell bodies of the TN-GnRH3 neurons (Whitlock et al., 2003; Gopinath et al., 2004; Whitlock, 2004). In zebrafish, the GnRH3 receptor is present in the adult OE as assayed by immunocytochemistry (Whitlock et al., 2006) and the isolation of GnRH3-receptor mRNA from olfactory organ tissues (Corchuelo et al., 2017). The presence of both the GnRH3 peptide and its receptor in the olfactory organ suggests potential modulation *via* an internal feedback loop or perhaps *via* exogenous interaction with GnRH peptides released from conspecifics as has been observed for other hormones (Stacey et al., 2003). The recent concept of a neurovascular unit (NVU) describes the relationship between neurons and blood vessels where the NVU incorporates cellular and extracellular components involved in regulating brain function (Schaeffer and Iadecola, 2021). Future studies are needed to determine whether the TN-GnRH3 system in the adult zebrafish (Figure 2A), and other fishes, forms a NVU with the blood vasculature in the peripheral olfactory organs or as suggested years ago (Zheng et al., 1988), as a novel neurohaemal organ, which would allow for internal and external interactions of neural circuits. Thus the GnRH3 neuromodulatory neurons of the TN and their fibers have a close association with the olfactory tract and blood vasculature making them well situated to modulate olfactory-mediated behaviors in response to alterations in the external environment.

## Olfactory-Immune Link Underlying Behaviors

Studies in mice and fish have shown that olfactory-mediated behaviors are mediated by major histocompatibility complex (MHC) peptides where they act as olfactory cues underlying mate choice decisions (Boehm and Zufall, 2006). Furthermore, documented interactions between the MHC, olfactory receptors, and T cell antigen receptors may underlie the selection of potential mates based on genetic suitability and health as well as promote the evasion of pathogens and predators (Tizard and Skow, 2021). Previously we have shown that zebrafish can make and maintain memories of odors experienced as juveniles and that this “olfactory imprinting” is associated with transcriptional changes within the olfactory organs (Harden et al., 2006; Whitlock, 2006; Calfun et al., 2016). The subsequent analysis of olfactory organ cell types led to the discovery of local neutrophils and macrophages/microglia in the olfactory organs of developing and adult zebrafish (Palominos and Whitlock, 2021; Palominos et al., 2021; Figure 2B). These observations build on previous data showing the intimate association between the olfactory sensory and immune systems with macrophages and/or microglia in cultures of olfactory mucosa (Pixley, 1996), the major histocompatibility complex class I (MHCI) in the olfactory placode of the mouse (Chacon and Boulanger, 2013), and recombination activating gene (RAG) gene expression in the intact OE of zebrafish (Jessen et al., 2001). Thus the process of forming an olfactory memory, such as that happens in olfactory imprinting, is tightly correlated with activating aspects of the immune system within the olfactory organs.

Olfactory imprinting involves environmental cues as observed in salmon migration to the natal stream (Scholz et al., 1976), the navigation behavior of coral reef fish to their home reef (Dixson et al., 2014), or the modification of alarm response (Wisenden, 2000), and is a plastic process that involves a variety of chemical signals. Yet imprinting is also important in discerning the relatedness among conspecifics and involves specific genetically determined kin signals communicated through MHC peptides (Gerlach et al., 2019). Previously, the role of the immune system has been shown to be important in kin recognition, suggesting a genetic predisposition to kin odor, as genes of the immune system (MHC) are the basis for urine-born peptides carrying information about “self” and “other” (Gerlach et al., 2008; Hinz et al., 2013).

Evidence for the importance of immune-based olfactory clues is also found in the mating strategies of stickleback fish where female sticklebacks evaluate male MHC-associated olfactory cues during the process of mate choice, choosing males that optimally complement the female’s MHC alleles to produce offspring with a population-specific optimal number (Andreou et al., 2017). The olfactory cues provided by the MHC complex in sticklebacks have now been shown to link olfactory assessment of mate choice with habitat-specific adaptation where habitat-specific immunogenetic diversity links habitat quality with individual qualities thus providing a mechanism for ecological speciation in vertebrates (Gahr et al., 2018). Taken together studies on the neuro-immune basis of olfactory mediated behaviors such as kin selection indicate that the olfactory tracts within the forebrain are activated, including the limbic system of these teleost fishes, by different subsets of immune-related peptides. The discovery of extensive immune structures in the olfactory organs suggests a potential role for circulating peripheral immune cells in the creation of odor-based memories that are essential for survival.

## Olfaction, Climate Change, and Future Evolution

### Anthropogenic Changes in Aquatic Environments

In considering the evolution of the teleost forebrain we must now look to the future, a future where it is becoming increasingly evident that the “olfactory ecosystem” of both marine and freshwater fishes is undergoing dramatic changes. The primary pathway for detecting odors is *via* the olfactory sensory epithelia. Because the olfactory neuroepithelium is in direct contact with the surrounding environment, it is potentially vulnerable to detrimental changes in aquatic ecosystems. Olfaction is an essential sensory system for fishes as they use odors to find food, safe habitats, avoid predators, recognize conspecifics as well as to find suitable spawning grounds. With climate change affecting aquatic environments, it is becoming apparent that water pollution and acidification driven by high levels of atmospheric CO_2_ may result in decreased behavioral responses to odorants and reduction in capacity for odor learning in many fish species. Thus these changes in the ability to perceive and react to odorants will have a profound effect on the survival of fishes, which make up half of the vertebrate biodiversity of the planet.

### Olfactory Learning

As part of learning to react to biotic factors in their environment, fishes show a wide variety of complex olfactory-mediated learning behaviors. One of the most dramatic examples of olfactory learning, as mentioned above, is olfactory imprinting where the salmon learn the scent of their home stream (freshwater) and replay this olfactory memory during their homing migrations from the ocean (Scholz et al., 1976; Ueda, 2012). Fishes also show a wide variety of complex olfactory learning behaviors involving two sensory functions such as the case of fathead minnows who learn to recognize predators through the pairing of predator scent with the sight of experienced minnows reacting with fear, a learning process that is not species-specific (Chivers and Smith, 1994; Wisenden et al., 1994; Gazdewich and Chivers, 2002). Interestingly, fish learn to pair both predator cues with the presence of alarm pheromone (von Frisch, 1938, 1941) so that they can respond more strongly to the release of the alarm pheromone (Levesley and Magurran, 1969; Brown and Smith, 1998).

We now know that CO_2_-induced acidification of freshwater systems affects juvenile freshwater phase of development in salmon (*Oncorhynchus gorbuscha*, *Salmo salar*) where they showed significant alterations in olfactory responses to alarm cues and amino acids (Moore, 1994; Ou et al., 2015). These are troubling results given that the anti-predator behaviors are affected and also potentially olfactory memory because amino acids in natal rivers are thought to play an important role in imprinting and homing migration (Shoji et al., 1994, 2000). Subsequent studies investigating the ocean phase migration of juvenile coho salmon (*Oncorhynchus kisutch*) and their responses to elevated CO_2_ revealed altered expression of neuronal signaling genes within the OBs and to a lesser extent the olfactory organs suggesting that elevated CO_2_ affects olfactory processing (Williams et al., 2019). Thus alterations in the external environmental conditions may interfere with olfactory function and compromise behaviors essential for the survival of fish and other aquatic species.

### Hot, Acidic, and Dirty

**Figure 3:**
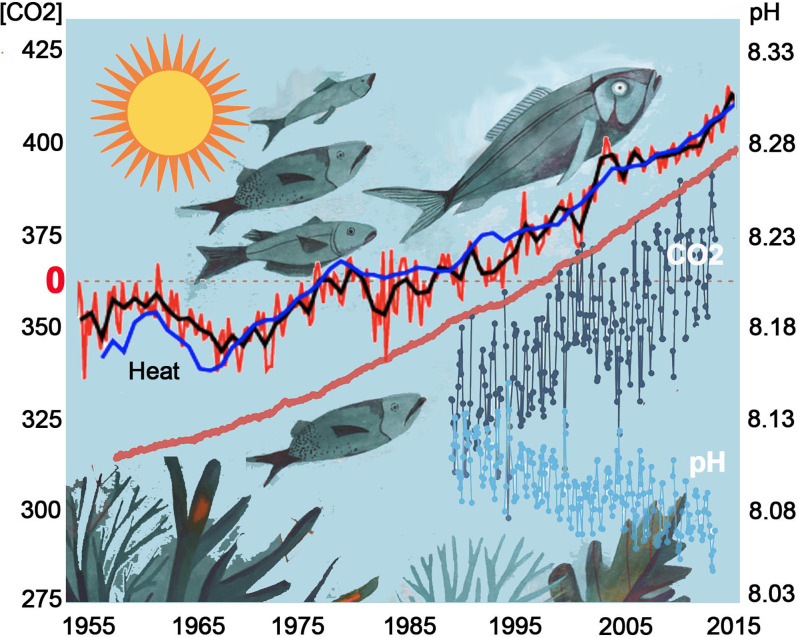
Ocean heating and increasing CO_2_ concentration affect fish behaviors. As the oceans heat up (Heat), marine species are moving deeper in the water column and towards the poles to escape rising temperature (Heat, increases since 1955 over average dotted line; https://www.ncei.noaa.gov/access/global-ocean-heat-content/#null). Concomitant increases in CO_2_ in the atmosphere (red), and oceans (gray), are driving the pH acidic (blue) thus affecting olfactory-mediated behaviors in marine animals. https://oceanacidification.noaa.gov/OurChangingOcean.aspx.

In the last 10 years, an increasing number of studies in both freshwater and marine ecosystems have alerted the world to the significant impacts of anthropogenic changes on our oceans (Garcia-Soto et al., 2021) such as acidification of aquatic ecosystems accompanied by ocean heating (Figure 3). Most notable are the effects on the olfactory abilities of fish where impaired behavioral responses to a variety of social odor cues will potentially result in abrupt changes in population dynamics and reduced complexity of community structures as evidenced by the effects on emblematic coral reef fishes (Munday et al., 2009, 2010; Heuer et al., 2016). As more studies examine impaired olfactory-mediated behaviors in freshwater and marine ecosystems, it is becoming apparent that the underlying mechanisms may be quite different. In acidified freshwater, molecular changes to chemical cues along with reduced olfaction sensitivity in the peripheral epithelia appear to be the primary causes of olfactory-mediated behavioral deficits (Leduc et al., 2013; Commentary: Porteus et al., 2021). In contrast, experiments simulating future ocean acidification suggest that high levels of CO_2_ can interfere with brain neurotransmitter function as the primary cause of olfactory-mediated behavioral deficits in fish (Nilsson et al., 2012; Leduc et al., 2013; Hamilton et al., 2014).

Worldwide, the increasing salinity of freshwater ecosystems due to anthropogenic activities is leading to ecosystem degradation and serious losses of biodiversity (Zhao et al., 2021). In freshwater systems, the animals are not immune to the effects of environmental degradation such as changes in salt concentrations due to drought or mining effluents. Recently it has been shown that juvenile zebrafish use a specific subset of OSNs, as opposed to taste receptors, to detect and avoid increasing salt concentrations (Herrera et al., 2021). This surprising result that suggests the olfactory epithelia is important in determining the salt concentration and thus habitable environments. Studies using juvenile rainbow trout showed that when pH is lowered (6.5) or when sodium salt concentrations are raised, the responses to standard olfactory cues are impaired. Furthermore, the pH and salts can modify contaminant-induced olfactory toxicity (Lari et al., 2019).

Previously copper present in runoff has been shown to affect olfactory sensitivity in salmon (Baldwin et al., 2003, 2010) and trigger an immune response within zebrafish olfactory epithelia (Palominos and Whitlock, 2021; Palominos et al., 2021). More recently another form of copper, nanoparticles (CuNPs), used in commercial applications such as anti-bacterial and anti-fungal agents in textiles and coatings, has been shown to impair fish olfactory function in rainbow trout. Analysis of olfactory organs showed that, unlike copper exposure, genes involved in olfactory transduction, neurogenesis, and immune response were all downregulated in the copper nanoparticle damaged olfactory mucosa (Razmara et al., 2021). This study presents yet another type of environmental contaminant that can impact olfactory-based survival of aquatic animals.

### Future Evolution

While many fish species are stenohaline, or unable to withstand wide variation in salinity, teleosts fish have clearly evolved to tolerate many unusual niches in distinctly different ecosystems. The existence of euryhaline fishes that can survive in saltwater, freshwater, and brackish water is living evidence of the evolution of salt tolerance (Evans and Kültz, 2020) and thus these fishes may be the most able to retain olfactory driven behaviors as the rate of climate change accelerates.

### Evolution of Olfactory Receptors in Fishes

Unlike terrestrial vertebrates, fishes lack a vomeronasal organ yet the teleost olfactory epithelia express all classes of tetrapod odorant receptors: main odorant receptors (ORs), trace amine-associated receptors (TAARs), vomeronasal receptors type 1 (V1Rs), and type 2 (V2Rs). In comparing the three types of G protein-coupled odorant receptors, the OR, V1R, and V2R, gene repertoires of teleost are smaller in size compared to mammalian species, yet show greater overall OR diversity as evidenced by a larger number of major clades (Alioto and Ngai, 2005; Korsching, 2009). In contrast, the gene repertoire of teleost TAARs is much greater than the corresponding mammalian genes with some fish species expressing over 100 functional isoforms (Gainetdinov et al., 2018). Analysis of odorant receptors in zebrafish, Medaka, stickleback, fugu, and spotted green pufferfish has revealed gene losses and gains in TAARs suggesting that genes coding for these different types of odorant receptors may be under lineage-specific adaptive evolution (Hashiguchi et al., 2008).

A recent analysis of the evolutionary divergence of ORs and their association with ecological adaptations in different species showed that Mariana snailfish, a fish found in the Mariana trench (6,000 m), has many fewer ORs and more OR pseudogenes compared to Tanaka’s snailfish a shallow-sea relative, with both species having similar numbers of TAARs (Jiang et al., 2019). This most likely reflects the rapid evolution of the OR repertoire in response to the unusual ecological niche of the deep-sea trenches. The suggestion that in relatively short spans of time ORs can emerge and become fixed is supported by the reported low proportion of orthologous receptors found in closely related species. The evolution of the OR genes most likely reflects the important chemical features of an animal’s ecological niche (Niimura and Nei, 2005; Adipietro et al., 2012; Bear et al., 2016).

Olfactory receptor repertoire might be one of the leading causes for fish sympatric speciation: *Coptodon* cichlid fishes found in Lake Ejagham in Africa have diverged at the same time that a cluster of olfactory genes had introgressed (Poelstra et al., 2018), suggesting olfaction as a causal trigger for fish adaptive radiation. Within domesticated fish populations, environmentally driven “olfactory” genomic plasticity has been noted. In the Senegalese sole, comparisons of transcript profiles from tissues of olfactory organs of “domesticated” fish species and their wild counterparts have uncovered distinct differences between cultured and wild animals in genes related to olfaction, reproduction, nutrient sensing, and immune system, revealing a genomic response to selection (Fatsini et al., 2016). Taken together studies on OR gene evolution support the possibility that the genome may accommodate environmental pressures in the future.

### Adaptation and Transgenerational Effects

In contrast to adaptation, which involves the selection of genetic variation that increases the fitness of the animal, acclimation relies on plastic responses in physiology, morphology, or behavior to a new environment such as the changes being imposed on aquatic environments as a result of climate change. Acclimation to ocean acidification has been reported in anemonefish (*Amphiprion melanopus*) where increased growth and survival in a high CO_2_ environment were observed in juveniles whose parents had been previously exposed to high CO_2_ (Miller et al., 2012; Munday, 2014). Offspring of parents living in acid waters (Allan et al., 2014) or under hypoxic conditions (Ho and Burggren, 2012) showed greater tolerance to the environmental stressors experienced by their parents. Effects of transgenerational plasticity have been observed in the Atlantic Cod (*Gaddus morhua*) where larval survival at elevated CO_2_ levels was increased if the parents were acclimated to the same CO_2_ exposure; yet with the caveat that these effects were seen only under conditions of high food availability, suggesting that transgenerational acclimation to excess anthropogenic carbon dioxide in ocean waters is dependent on the availability of surplus food (Stiasny et al., 2018).

The mitigation of negative effects of CO_2_ on growth and aerobic capacity by transgenerational acclimation in fishes to date does not appear to restore olfactory responses to alarm cues and other olfactory driven behaviors in juvenile spiny damselfish (*Acanthochromis polycanthus*, Welch et al., 2014). Furthermore, genomic approaches using reef fishes have identified transcriptional changes induced by elevated CO_2_ levels in within-generation treatments that returned to baseline levels in fish that were transgenerationally exposed to elevated CO_2_ levels (Schunter et al., 2018), indicating that the environmental-induced phenotype interacts with the parental phenotype as organisms attempt to respond to ocean acidification.

## Conclusions

Without doubt, the olfactory tracts of the forebrain and their targets in the limbic system play essential roles in monitoring sensory input, deciding on strategies, and modifying behaviors accordingly. Studies examining the effects of pH/CO_2_ on aquatic ecosystems have shown profound effects on the olfactory sensory system: changes in acid–base regulation under elevated CO_2_ affects the functioning of gamma-aminobutyric acid-mediated (GABAergic) neurotransmission and acidic pH values can directly affect the protonation, and charge distribution of odorants and/or their receptors (see for comment: Porteus et al., 2021). Whether acclimatization and adaptation can have long-lasting effects on the persistence of a species is an answer that lies in the future. More studies are needed, as suggested here, to better understand the potential roles of both the olfactory-neuromodulatory and olfactory-immune pathways in the survival of fishes. We imagine both scientists and non-scientists who are concerned about the rapid loss of biodiversity driven by anthropogenic forces are interested in the role of behavioral flexibility and the underlying genomic plasticity as teleosts and all other species struggle to adapt to a rapidly changing world.

*“I can get a clear picture of any face I feel like remembering, and I can hear whatever Beethoven quartet I want to recall, but except for the leaf bonfire I cannot really remember a smell in its absence*.”

*—Lewis Thomas (1985)*.

## Author Contributions

KEW prepared the figures and wrote the initial text. MFP revised the text and figures, including the final version. All authors contributed to the article and approved the submitted version.

## Funding

This work was supported by Grants/Fellowships Fondo Nacional de Desarrollo Científico y Tecnológico (FONDECYT) 1160076 (KEW); ICM-ANID Instituto Milenio Centro Interdisciplinario de Neurociencias de Valparaíso PO9-022-F (MFP), supported by the Millennium Scientific Initiative of the Ministerio de Ciencia (KEW and MFP); CONICYT Doctoral Fellowship (ANID) 21161437 (MFP). The funding bodies did not take part in the design of the study, the collection, analysis, and interpretation of data, or in the writing of the manuscript.

## Conflict of Interest

The authors declare that the research was conducted in the absence of any commercial or financial relationships that could be construed as a potential conflict of interest.

## Publisher’s Note

All claims expressed in this article are solely those of the authors and do not necessarily represent those of their affiliated organizations, or those of the publisher, the editors and the reviewers. Any product that may be evaluated in this article, or claim that may be made by its manufacturer, is not guaranteed or endorsed by the publisher.
