# Corrigendum: The olfactory tract: Basis for future evolution in response to rapidly changing ecological niches

**DOI:** 10.3389/fnana.2023.1153062

**Published:** 2023-03-23

**Authors:** Kathleen E. Whitlock, M. Fernanda Palominos

**Affiliations:** ^1^Centro Interdisciplinario de Neurociencia de Valparaíso (CINV), Universidad de Valparaíso, Valparaíso, Chile; ^2^Instituto de Neurociencia, Universidad de Valparaíso, Valparaíso, Chile

**Keywords:** gonadotropin releasing hormone (GnRH), immune system, neutrophils, climate change, limbic system, teleost fishes

In the published article there was a missing citation in Figure 1. Figure 1A was adapted from Calvo-Ochoa and Byrd-Jacobs (2019). The corrected Figure 1 caption is below.

Figure 1. The connections from the peripheral olfactory epithelia to the olfactory bulbs are highly conserved in vertebrates. In both in teleost fish (**A**, zebrafish: modified from -Calvo-Ochoa and Byrd-Jacobs, 2019) and humans **(B)** the OSNs relay information to the olfactory bulbs (blue) continuing to the dorsal pallium in fishes **(A)**, and the olfactory cortex/lateral pallium **(B)** in mammals, thus bypassing the thalamus (orange). Both species have projections from the olfactory bulbs (blue) to the amygdala (red, **B**) and its proposed equivalent in teleosts, the dorsomedial pallium (red, **A**).

The authors apologize for this error and state that this does not change the scientific conclusions of the article in any way. The original article has been updated.
